# Using Intervention Mapping to Develop a Virtual Reality Intervention to Support COVID-19 Vaccination Among Black Families

**DOI:** 10.1177/29941520251377072

**Published:** 2025-09-12

**Authors:** Francis J. Real, Andrea Meisman, Julie Wijesooriya, Lori E. Crosby, Brittany L. Rosen

**Affiliations:** ^1^ Department of Pediatrics, University of Cincinnati College of Medicine, Cincinnati, Ohio, USA.; ^2^ Division of General and Community Pediatrics, Cincinnati Children’s Hospital Medical Center, Cincinnati, Ohio, USA.; ^3^ Division of Adolescent and Transition Medicine, Cincinnati Children’s Hospital Medical Center, Cincinnati, Ohio, USA.; ^4^ Center for Clinical & Translational Science & Training, Cincinnati Children’s Hospital Medical Center, Cincinnati, Ohio, USA.; ^5^ Division of Behavioral Medicine and Clinical Psychology, Cincinnati Children’s Hospital Medical Center, Cincinnati, Ohio, USA.

**Keywords:** virtual reality, community-based participatory research, community advisory board, vaccine hesitancy, COVID-19 vaccine

## Abstract

Pediatric uptake of COVID-19 vaccines is low, particularly among Black children. There is a critical need for pediatric clinicians to address COVID-19 vaccine concerns through community-centered, culturally appropriate communication strategies. Virtual reality (VR) may provide a modality to support clinicians’ relational skills related to COVID-19 vaccine by providing a safe, realistic environment to practice preferred communication behaviors. The aim of this project was to use Intervention mapping (IM), a six-step framework, to cocreate a VR intervention with Black families to support COVID-19 vaccine uptake. For IM, step 1 (develop a logic model of the problem) and step 2 (identify program objectives and outcomes), our team used quantitative and qualitative methods to identify caregiver, adolescent, and young adult factors associated with intention and decisions to vaccinate against COVID-19. IM step 3 (program design) consisted of assembling planning board sessions with the Black community stakeholders including adolescents, young adults, and caregivers, along with primary care pediatric clinicians. A modified Delphi technique was used to build consensus for salient COVID-19 vaccine concerns and communication preferences. During IM step 4 (program production), the VR intervention (Virtual Immersive Communication Training on Recommending Immunization COVID-19) and accompanying simulation flowsheets were developed. An implementation plan was developed for IM step 5 (program implementation) and an evaluation plan for IM step 6 (evaluation). Through IM, an intervention centered on the needs and preferences of Black families was successfully developed. Lessons learned using IM included: (1) the importance of collaborating with patients and families on clinical topics where there is limited data, (2) the use of rigorous methods such as the modified Delphi approach to help build consensus, and (3) community relationships are critical to drive culturally informed technology-based interventions, especially when urgency is required.

## Introduction

While the COVID-19 vaccine is effective at preventing severe disease^1^ and hospitalization^2^ in pediatric populations, only 7% of children, aged 6 months to 17 years were reported to be up-to-date with the COVID-19 vaccine as of January 8, 2025.^3^ Furthermore, during the 2021/2022 season, COVID-19 was the 8th leading cause of death in children and adolescents, outranking both influenza and pneumonia.^4^ Though COVID-19 vaccination is low across all sociodemographic subgroups, Black children have lower COVID-19 vaccination rates when compared to White and Hispanic children.^5^ This may in part be due to a lack of culturally informed interventions that support vaccination, as individuals of different racial and ethnic backgrounds may have reasons for hesitancy distinct from the general population due to historical exploitation and mistrust.^6^ Lower vaccination rates may contribute to the disproportionately higher COVID-related morbidity and mortality among Black children.^7^ Among racial minority groups, rapid development of the vaccine and government mistrust have surfaced as prevalent concerns leading to vaccine hesitancy.^8,9^ Identification of culturally relevant communication strategies to overcome vaccine hesitancy and support the uptake of the COVID-19 vaccine is critical to improving pediatric health outcomes.^6,10,11^

### COVID vaccine recommendation

Strong and consistent vaccine recommendations from health care providers have been found to be a powerful tool to counter vaccine concerns.^12–19^ Specifically, introducing a vaccine in a presumptive approach, using short statements indicating vaccination as the default option (e.g., “The patient is due for vaccines today”), has demonstrated effectiveness at increasing uptake of standard childhood vaccines as well as human papillomavirus (HPV) and influenza vaccines.^20–24^ Based on this evidence, the Centers for Disease Control and Prevention and other professional organizations recommended the use of a presumptive approach by health care providers as a strategy to increase COVID-19 vaccine uptake.^25,26^ However, the American Academy of Pediatrics urges caution in using the presumptive approach indiscriminately, as it limits opportunities to provide culturally relevant vaccine information that may be necessary to facilitate trust.^27^ Given the surge in misinformation and polarized vaccine discourse, counseling on the COVID-19 vaccine may require nuanced behaviors distinct from historically identified counseling practices. For instance, motivational interviewing (MI)—a patient-centered communication style to build rapport and support collaborative decision-making^28^—may play a role in supporting COVID-19 vaccine counseling as it has demonstrated effectiveness in addressing hesitancy around the HPV and influenza vaccines.^29,30^

### Intervention mapping

Intervention mapping (IM) is an established collaborative process incorporating input from key stakeholders to develop an intervention. IM uses a six-step approach to integrate theory, empirical findings, and information from the target population. The sequential six-step process includes identifying a logic model of the problem (step 1), establishing program outcomes and objectives (step 2), designing a program (step 3), producing the program (step 4), developing an implementation plan (step 5), and evaluating the program (step 6; Table 1).^31^ IM methods have been previously employed to cocreate interventions with community members and providers to increase vaccine uptake including within urban pediatric clinics and federally qualified health centers.^34,35^ IM was also used to develop and adapt a storytelling intervention targeting HPV vaccine uptake among Hispanic adolescents.^36^ Thus, IM has demonstrated the capacity to successfully engage stakeholders in intervention development related to vaccination. However, it has not been applied to virtual reality (VR) interventions targeting pediatric clinician communication, specifically among racial minoritized populations, to promote COVID-19 vaccination.

**Table 1.** Intervention Mapping Steps, Methods, and Outcomes for COVID-19 Vaccine Virtual Reality Intervention^31^

Timeline	Intervention mapping steps and methods	Outcomes
Online survey: January 2021 Interviews: December 2021–February 2022	Step 1: Develop a logic model of the problem • Adolescents, young adults, and caregivers recruited to complete an online survey to assess knowledge, attitudes, and intentions to receive COVID-19 vaccine^32^ • Interviews conducted with caregiver-adolescent dyads to further understand acceptance of the COVID-19 vaccine^33^	• Facilitators and barriers associated with COVID-19 vaccine intention • Logic model of COVID-19 vaccine hesitancy
Introduction session to WE-CRAB: October 2021	Step 2: Identify objectives and outcomes • Guided by the *logic model of the problem* (step 1), we used a brainstorming session with our primarily Black and female planning board, led by a facilitator, to develop curricular change objective and performance objectives	• Logic model of change to guide program development
Community planning board sessions 1–4: January 2022–February 2022 Clinician planning board sessions: February 2022–March 2022	Step 3: Design the program • Using the *logic model of change* (step 2), we used a modified Delphi technique and design sessions with the planning board to identify: (a) salient sources of COVID-19 vaccine hesitancy for Black individuals, (b) messaging from clinicians to address determinants with Black families	• Salient sources of hesitancy • Messaging for clinicians to address COVID-19 vaccine hesitancy
Community planning board session 5: March 2022	Step 4: Program production • Based on the design sessions, we developed the virtual reality simulation scenarios and flowsheets, training rubrics, and tangible educational materials. • We developed a prototype of the virtual reality curriculum to review with the planning board for feedback	• Tangible educational materials (e.g., sample language) for virtual reality training
	Step 5: Develop implementation plan • We identified potential program barriers to implementation using the reach, effectiveness, adoption, implementation, maintenance (RE-AIM) planning questions worksheet.	• Implementation plan addressing potential barriers
Usability testing of VICTORI-COVID19: August 2022 VICTORI-COVID-19 Efficacy pilot: September 2022–January 2023	Step 6: Evaluation • Using the program developed in steps 3 and 4 as well as the implementation plan developed in step 5, we implemented the VR simulations to train health care clinicians in COVID-19 vaccine counseling and assess COVID-19 vaccination rates over time in a clinical setting. • Also, we plan, in the future, to assess the impact of the intervention on Black families’ trust in their health care provider.	• Effectiveness data to assess the virtual reality intervention’s impact on COVID-19 vaccine rates

VICTORI, Virtual Immersive Communication Training on Recommending Immunization; WE-CRAB, West End Community Research Advisory Board.

### Virtual reality intervention

VR is a computer-generated environment where a user interacts with a digital environment and digital characters in an authentic and realistic manner. VR interventions can be delivered via a 3D-mounted headset or screen-based.^37^ Prior studies have demonstrated the capacity for brief VR interventions to enhance communication competencies, specifically MI skills in providers.^29,30,38^ This is notable as provider training in MI is often lengthy (5–18 h) including workshop or lecture-based formats that may be insufficient to impart these advanced communication competencies.^39^ Our team has previously developed VR-based interventions aiming to train providers in evidence-based communication.^29,30,40^ These interventions entitled Virtual Immersive Communication Training on Recommending Immunizations (VICTORI) have demonstrated a positive impact on influenza and HPV vaccination rates.^29,30^ Within VICTORI, a facilitator screen shares a virtual clinic space with a provider through a teleconferencing software. The virtual clinic space includes a graphical caregiver and child that respond in real time to verbal prompts from the provider. The facilitator drives the characters’ verbal and nonverbal responses based on a provider’s specific behavior to reinforce the use of evidence-based communication. Following participation in a specific scenario, a provider receives immediate feedback from the facilitator regarding their performance with an opportunity to repeat cases until demonstrating skill is mastered. Thus, VICTORI may overcome challenges of traditional training methods by allowing for deliberate practice^41^ of communication skills in a safe, immersive environment supporting behavior change.

### Purpose

Though the VR intervention platform provides an ideal environment for practicing communication behaviors relevant to COVID-19 vaccine counseling, there is a lack of evidence-based communication strategies to support COVID-19 vaccine uptake among racially minoritized populations. Thus, we sought to adapt the VICTORI intervention to focus on COVID-19 vaccine counseling among Black pediatric populations using IM.^31,36,42,43^ The specific objectives included identifying the most relevant sources of COVID-19 vaccine hesitancy, establishing consensus on preferred counseling strategies, and obtaining feedback on storyboards of VR scenarios among a planning board representative of the priority population. By using culturally responsive approaches, interventions may address the social and cultural needs of the patient, promoting positive patient-provider interactions when discussing the COVID-19 vaccine.^44^

## Methods

This study occurred at a Midwestern academic medical center and within an urban community setting. It was approved by the Cincinnati Children’s Hospital Medical Center Institutional Review Board (2022–0046). The academic medical center’s adolescent medicine primary care clinic was the target site for future implementation. This clinic completes approximately 5,000 primary care patient visits annually. Of the patients receiving care at the clinic, an estimated 72% of the patients are Black. Adaption of the intervention occurred in the winter of 2021 through the spring of 2022. At that time, an emergency use authorization (May 10, 2021) for the Pfizer-BioNtech COVID-19 vaccine had been expanded to include adolescents aged 12–15 years, followed by authorization for those ages 5–11 years old (October 29, 2021).^45^ In addition, the Delta and Omicron variants of COVID-19 were circulating throughout the United States, with nearly 1 million daily infections reported in January 2022.^46^ Thus, the project was timely to meet the needs of the community.

### Planning board participants

To ensure the intervention would be patient-centered as well as appropriate and feasible to providers, we established two planning boards: (1) community and (2) clinician.^47,48^ To establish the community planning board, we collaborated with the medical center’s community engagement consultant. The consultant served as the connector to a community research advisory group, West End Community Research Advisory Board (WE-CRAB). WE-CRAB consists of community stakeholders who engage, guide, and collaborate with researchers to support the health goals of the West End neighborhood, located in Cincinnati, Ohio. We had an introductory meeting with WE-CRAB to present our project’s goals, objectives, processes, and outputs. Next, we gauged WE-CRAB’s interest in collaborating. After confirming interest, we established the community planning board with those members of WE-CRAB as well as additional identified community members whose interests were most aligned with the project. The community and research team indicated a preference for virtual planning board meetings for ease of attendance and to align with social distancing recommendations at the time. Prior to participation in the planning board, community and clinician participants indicated consent or assent as appropriate based on the age of the member via *an* online database. Additionally, at the first community planning board sessions, the facilitators reviewed once again the study information. The participating community members were predominantly Black (*n* = 15; 94%), non-Hispanic (*n* = 16; 100%) and female (*n* = 15; 94%) and included adolescents (*n* = 6; age range 13–17), young adults (*n* = 4; age range 20–21), and caregivers (*n* = 6; age range 33–63).

To establish the clinician planning board, we invited clinicians working at a large urban pediatric primary care clinic via email. This clinic was specifically selected as it was located at the same Midwestern academic medical center as the target adolescent medicine clinic and also provided primary care for adolescent patients. Importantly, 75% of patients seen at this primary care clinic identify as Black or African American. The clinician planning board included a total of five clinicians (4 physicians and 1 nurse) with varying demographics, including Black (*n* = 2; 40%), non-Hispanic (*n* = 5; 100%), and males (*n* = 1; 20%). The nurse participant had 11–15 years of experience. The attending physician participants had a range of experience in their current role including 1–2 years (*n* = 1), 3–5 years (*n* = 1), 6–7 years (*n* = 1), and >15 years (*n* = 1). A summary of the IM steps is included in Table 1.

### Intervention mapping step 1

**FIG. 1.**
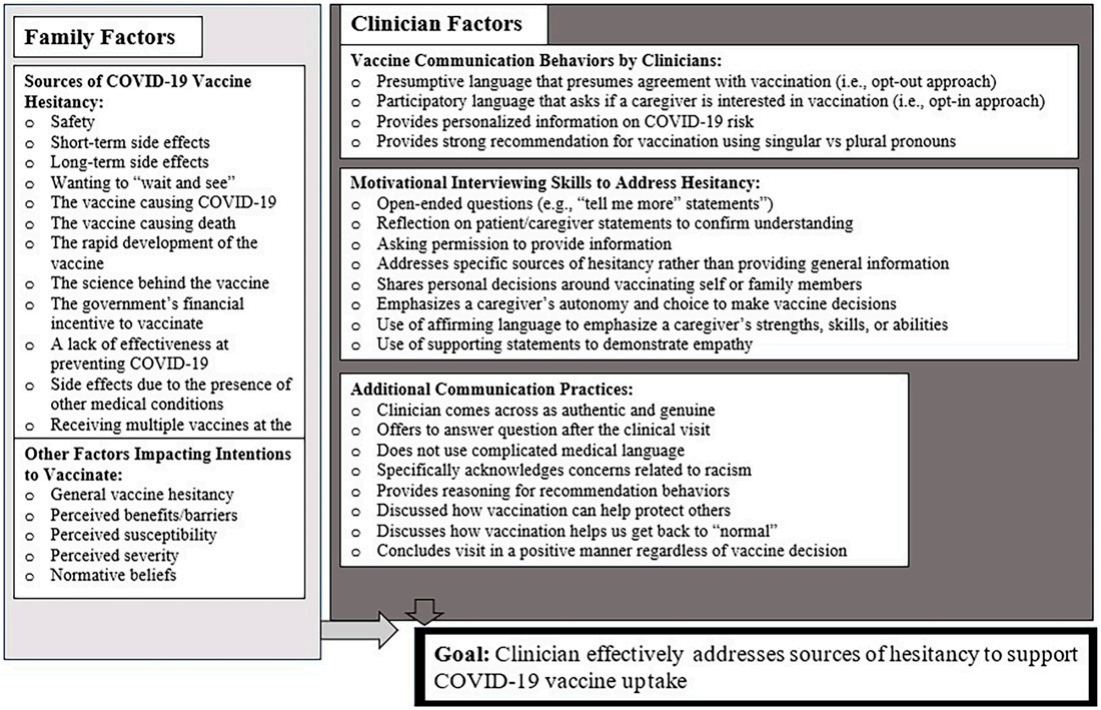
Logic model of the problem that highlights items included within the modified Delphi.

Prior to the planning boards, we conducted mixed-methods studies assessing adolescents, young adults, and caregivers’ determinants associated with COVID-19 vaccine intentions to complete IM step 1 (develop logic model of the problem).^32,33^ In January 2021, we conducted a cross-sectional survey of adolescent (*n* = 242; mean age [standard deviation (SD)] =14.8 [1.8] years) young adults (*n* = 333; mean age [SD] = 20.4 [2.0] years), and caregivers (*n* = 563; mean age [SD] = 35.5 [8.9] years) to identify variables associated with intention to receive a COVID-19 vaccine. Participants were recruited via email from primary care clinics and the medical center’s Office of Clinical and Translational Research. The study found that variables associated with intention to receive the COVID-19 vaccine included general vaccine hesitancy, perceptions about COVID-19 vaccine safety, and normative beliefs regarding the COVID-19 vaccine.^32^ Next, we explored factors influencing caregiver and adolescents’ COVID-19 vaccine decision-making during the Omicron surge in winter 2021–2022 via qualitative interviews within a large, urban pediatric primary care clinic. Participants identified the perceived risk of COVID-19, the opinions of other family members, and two-way communication with clinicians as factors influencing their COVID-19 vaccine decisions.^33^ From these data, we developed a logic model of the problem (Fig. 1) encompassing sources of COVID-19 vaccine hesitancy, other factors impacting intentions to vaccinate, potential communication strategies to overcome hesitancy, and training strategies for clinicians.

### Intervention mapping step 2

Our primary outcome, based on the population’s needs, was to improve COVID-19 vaccine uptake in the pediatric population. The secondary outcome was to increase clinicians’ skills in using patient-centered vaccine counseling when addressing COVID-19 vaccine hesitancy. Aligned with these outcomes and driven by the determinants identified in step 1, we created program objectives focused on understanding how clinicians should ideally: (1) provide a COVID-19 vaccine recommendation, (2) address COVID-19 vaccine concerns and hesitancy, and (3) build trust with patients and caregivers.

### Intervention mapping step 3

To address the identified determinants and accomplish the program objectives, we selected VR as the educational strategy. The VR training facilitates deliberate practice, a personal and goal-oriented approach to skill development derived from Ericsson’s Theory on Expertise.^41^ This active learning process is characterized by engaging in the task or behavior (e.g., introducing the vaccine and addressing hesitancy), followed by immediate feedback regarding areas for improvement provided by a human facilitator, and then repeating the task, but changing behavior based on feedback.^29,30,38^ Thus, a facilitator confirms a learner has mastered specific behavioral objectives prior to completing the training. Thus, VR may provide substantial benefits over standard training strategies (e.g., didactic learning, workshops, and direct clinical observation).^39^

#### Delphi round 1

To adapt the VICTORI VR intervention to address COVID-19 vaccine hesitancy, we engaged in IM step 3—design a program—using a modified Delphi approach with the community and clinician planning boards, respectively.^49,50^ This approach is frequently used in health profession education to inform training on complex topics.^51,52^ We used a structured survey of factors identified in IM step 1 to inform the modified Delphi instrument including: (1) patients and caregivers’ potential vaccine concerns, (2) clinicians’ language to introduce the vaccine, and (3) MI skills for clinicians to address hesitancy. Planning board members were instructed to vote on keeping, removing, or modifying each of the items or state if they had no opinion.^50^ For each item, planning board members were provided with an option to provide free-text responses to support their decision or suggest item revisions. Responses were analyzed by the authors (F.J.R., A.M., and B.L.R.) and consensus was set at 70% to consider intervention inclusion. For the items that did not reach consensus, we reviewed any comments and suggested revisions and either removed the item or revised to better reflect the community insights. These items were included in the round 2 survey.

#### Delphi round 2

We presented the results of round 1 to the community planning board members to support transparency and answer questions regarding item revisions. Next, planning board members completed the second survey. The analysis and revision process from round 1 was repeated and produced a final list of recommended intervention components.

Of note, the clinician planning board completed only a single survey round that was informed by the results of the community planning board survey results.

#### In-person consensus meeting

We presented the results of round 2 to the planning board members, addressing any questions regarding the kept and removed items. The subsequent planning board sessions included the members viewing video demonstrations of a clinician using the skills identified in the Delphi method to introduce and address COVID-19 vaccine hesitancy during a clinical encounter to inform final decisions regarding the training content. Planning board members also provided feedback on exemplar language to be included in the VR training.

## Results

The results of the modified Delphi process are included in Table 2. There was a strong preference among planning board members for introducing the vaccine in a participatory approach, using open-ended questions to invite a caregiver to voice an opinion on vaccination (e.g., “Would you be interested in getting the COVID-19 vaccine today?”). This was in opposition to the previously described presumptive format that uses short statements indicating vaccination as the default option (e.g., “You are due for the COVID-19 vaccine today.”). The following COVID-19 vaccine concerns were identified via this consensus-building process as most important to Black families: (1) safety and side effects, (2) timeline of vaccine development, (3) long-term side effects, and (4) lack of efficacy due to breakthrough cases and need for boosters. Members identified the following MI and other relevant communication skills as the most important strategies to counter mistrust and validate the lived experience of Black families: (1) asking permission before providing education as a sign of respect, (2) providing information without medical jargon (i.e., being transparent using understandable language), (3) providing a recommendation for the vaccine using data (i.e., valuing the family’s ability to understand the rationale for the recommendation), and (4) openness to continue discussions if patient declines (i.e., respecting the family’s decision and process) (Table 3).

**Table 2.** Modified Delphi Survey Results

	Community Delphi round 1 (*n* = 15)			Community Delphi round 2 (*n* = 14)			Clinician Delphi round 1 (*n* = 5)		
	Week of January 10, 2022			Week of January 17, 2022			Week of January 24, 2022		
Timeline of survey completion	Keep item	Modify item	Remove item	Keep item	Modify item	Remove item	Keep item	Modify item	Remove item
Item	*n* (%)			*n* (%)			*n* (%)		
Sources of COVID-19 vaccine hesitancy									
Concerns about multiple vaccines at one visit (flu and COVID-19)	9 (60)	3 (20)	3 (20)	8 (57)	3 (21)	3 (21)			
Concerns regarding vaccine safety	**12 (80)**	2 (13)	1 (7)				**5 (100)**	0 (0)	0 (0)
Concerns regarding the short-term side effects from the vaccine (for example, body aches, chills, high temperature, headache)^a^	**11 (73)**	4 (27)	0 (0)				**5 (100)**	0 (0)	0 (0)
Concerned about the unknowns regarding long-term side effects from the vaccine^a^	**13 (87)**	2 (13)	0 (0)				**4 (80)**	1 (20)	0 (0)
Wanting to “wait and see” before getting the vaccine	9 (60)	4 (27)	2 (13)						
Concerns about the vaccine causing COVID-19	8 (53)	3 (20)	4 (27)	9 (64)	1 (7)	4 (29)			
Concerns about the vaccine causing death	11 (73)	3 (20)	1 (7)				3 (60)	0 (0)	2 (40)
Concerns that the vaccine was developed too fast^a^	**13 (87)**	1 (7)	1 (7)				**5 (100)**	0 (0)	0 (0)
Concerns about the science behind the vaccine	10 (67)	3 (20)	2 (13)						
Concerns about government’s financial incentives for people to get the vaccine	**12 (80)** ^b^	0 (0)	3 (20)				3 (60)	0 (0)	2 (40)
Concerns that the vaccine does not work	**11 (73)** ^c^	4 (27)	0 (0)						
Concerns that the vaccine does not work at preventing COVID-19 (for example, people still get “break-through cases”)^a^				**12 (86)**	2 (14)	0 (0)	**5 (100)**	0 (0)	0 (0)
Concerns that the vaccine does not work because we need a booster^a^				**10 (71)**	3 (21)	1 (7)	**4 (80)**	0 (0)	1 (20)
Concerns about side effects from the vaccine because of other medical conditions	**11 (73)** ^b^	3 (20)	1 (7)				3 (60)	0 (0)	2 (40)
Vaccine communication behaviors by clinicians									
Doctor offers vaccine as an option by stating, “You/your child are eligible for the COVID-19 vaccine. Would you be interested in getting vaccinated today?”^a^	**11 (73)**	2 (13)	2 (13)	**10 (71)** ^d^			3 (60)^d^		
Doctor assumes you are ready to vaccinate by stating, “You/your child are/is due for the COVID-19 vaccine.”	6 (40)	3 (20)	6 (40)						
Doctor brings up the topic of the vaccine by asking, “Now, have you/has your child gotten the COVID-19 vaccine yet?”	**13 (87)**	1 (7)	1 (7)	4 (29)^d^			2 (40)^d^		
Other factors impacting intentions to vaccinate									
Doctor provides information about you/your child’s risk if you have not received the vaccine by stating, “You/your child have/has asthma and are at a higher risk for severe illness and hospitalization if you are infected with COVID-19.”^e^	**11 (73)** ^c^	4 (27)	0 (0)				**5 (100)**	0 (0)	0 (0)
Doctor provides information about your child’s risk if they have not received the vaccine by stating “Because you/your child has asthma, you/your child is at a higher risk for severe illness/hospitalization if you/ s/he gets COVID-19. The vaccine can not only protect Alex from getting COVID-19 but also decrease the severity of illness and the possibility of hospitalization.”^e^				**12 (86)**	2 (14)	0 (0)			
Doctor provides strong recommendation for the COVID-19 vaccine by stating, “I strongly recommend the COVID-19 vaccine.”	**12 (80)**	2 (13)	1 (7)				**5 (100)**	0 (0)	0 (0)
Doctor provides strong recommendation for the COVID-19 vaccine from the medical community by saying, “We strongly recommend the COVID-19 vaccine.”	10 (67)	4 (27)	1 (7)						
Motivational interviewing skills to address hesitancy									
When you show concern or hesitancy about the COVID-19 vaccine, doctor asks questions about your concerns. For example, doctor says, “What specific concerns do you have about the vaccine?”^a^	**13 (87)**	2 (13)	0 (0)				**5 (100)**	0 (0)	0 (0)
After you provide specific concerns (for example, long-term side effects) about the COVID-19 vaccine, the doctor shows they understand your concerns by stating, “I hear that you are worried about the risk of long-term side effects.”^a^	**13 (87)**	2 (13)	0 (0)				**4 (80)**	0 (0)	1 (20)
After you provide specific concerns (for example, long-term side effects) about the COVID-19 vaccine, the doctor gives additional insights, “I hear that you are worried about the risk of long-term side effects and that can be scary. You just want to make the best choice for your/your child’s health.”^a^	**12 (80)**	2 (13)	1 (7)				**5 (100)**	0 (0)	0 (0)
Doctor asks permission to share information to address concerns and hesitancy about the COVID-19 vaccine before providing explanations and data by stating, “Is it alright if I share what I know about the COVID-19 vaccine with you?”^a^	**11 (73)**	4 (27)	0 (0)				**5 (100)**	0 (0)	0 (0)
Doctor emphasizes your choice as it pertains to what you choose for yourself and your health care by stating, “Your/Your child’s overall health is important to me and I strongly recommend this vaccine. I respect that ultimately it is your decision.”^a^	**13 (87)**	2 (13)	0 (0)				**4 (80)**	0 (0)	1 (20)
Additional Communication Practices									
Doctor answers your specific concerns.^a^	**14 (93)**	1 (7)	0 (0)				**5 (100)**	0 (0)	0 (0)
Doctor addresses your specific concerns by providing a personal example by saying, “Everyone in my family got vaccinated, including my parents, siblings, spouse, and children.” For parents ““I got all my children, who are aged 11, 13, and 17 years old, vaccinated against COVID-19.”	5 (33)	6 (40)	4 (27)						
Doctor asks if you have had any family members or close friends who have been impacted by COVID-19 to better understand your experience (e.g., being in the hospital, dying).^a^	**11 (73)**	3 (20)	1 (7)				**5 (100)**	0 (0)	0 (0)
Doctor shows awareness of your concerns by stating, “I appreciate you talking about the vaccine with me. You have clearly given this a lot of thought.”^a^	**12 (80)**	2 (13)	1 (7)				**5 (100)**	0 (0)	0 (0)
Doctor shows compassion and sympathy by stating, “I recognize that this is a hard decision.”	**13 (87)**	1 (7)	1 (7)				**5 (100)**	0 (0)	0 (0)
Doctor comes across as authentic and genuine during visit.^a^	**14 (93)**	1 (7)	0 (0)				**4 (80)**	1 (20)	0 (0)
Doctor offers to answer additional questions or concerns once you leave the office/clinic.	**14 (93)**	0 (0)	1 (7)				**5 (100)**	0 (0)	0 (0)
Doctor does not use complicated medical language when explaining the vaccine.^a^	**12 (80)**	2 (13)	1 (7)				**4 (80)**	1 (20)	0 (0)
Doctor explicitly acknowledges and validates racism within medicine by stating, “Racism is a powerful force in our country. It is not fair how people of color have been treated in medicine.”	6 (40)^c^	4 (27)	5 (33)						
Doctor explicitly acknowledges and validates racism within medicine and how getting the vaccine is a small act in fighting racism in medicine and states, “I want to make it my duty as a health care provider to work against racism within medicine.				4 (29)	3 (21)	7 (50)			
Doctor explains the reason behind their recommendation for the vaccine by stating, “I am recommending the vaccine because this will help protect you/your child from having severe COVID-19 and needing to go to the hospital.”^a^	**13 (87)**	2 (13)	0 (0)				**4 (80)**	0 (0)	1 (20)
Doctor explains how vaccinating is a way to help protect those around you/your child, such as family and friends.^e^	10 (67)	4 (27)	1 (7)	**11 (79)**	2 (14)	1 (7)			
Doctor explains how vaccinating is a way to end the pandemic and get back to “normal life”.	7 (47)	5 (33)	3 (20)						
Doctor respectfully addresses your decision to not vaccinate by stating, “I understand and respect that you/your child not want to be vaccinated today. This is a very important topic to me and I want you to know that I care about your/your child’s overall health and would like to talk about the vaccine at the next visit.”	9 (60)^c^	4 (27)	2 (13)						
Doctor respectfully addresses your/ parent’s decision to not vaccinate by stating, “I’m here to answer your questions and concerns about vaccines if your decision [for your child] changes. At you/your child’s next visit, I hope we can reevaluate if you are open.”^a^				**12 (86)**	0 (0)	2 (14)	**4 (80)**	1 (20)	0 (0)

Community Planning Board Round 1 was *n* = 15 due to a missing survey from *n* = 1; Community Planning Board Round 2 was *n* = 14 due to a missing survey from *n* = 2; Phrases you/your child were used interchangeably depending on if survey questions were posed to adolescent, young adult, or caregiver planning board members.

**Bold** denotes consensus >70% to keep the item by planning board members.

^a^ Denotes final item included in VICTORI-COVID19.

^b^ Denotes discrepancy between community and clinicians regarding the item in which discussion with planning boards determined if item should be included in the final intervention.

^c^ Denotes items that were reworded for subsequent rounds.

^d^ Denotes items in which planning board members were asked to choose a preferred COVID-19 vaccine introduction strategy without a response option to modify or remove.

^e^ Items were removed from the final intervention due to a lack of evidence supporting these statements at the time of intervention deployment.

**Table 3.** Recommendation and Motivational Interviewing Skills Included in the VICTORI-COVID19 Curriculum

Skill	Definition
Recommendation behaviors	
Introducing the COVID-19 vaccine using a **participatory approach**	Exploring a caregiver’s opinion on vaccination rather than presuming agreement to vaccinate (e.g., “How do you feel about getting your child the COVID-19 vaccine today?”)
Providing a **recommendation** for vaccination	Providing a recommendation without qualifying language regarding the strength of the recommendation (e.g., “I recommend the COVID-19 vaccine” rather than “I strongly recommend the COVID-19 vaccine.”)
**Accepting** a caregiver’s vaccine decision respectfully and leaving the door open for future vaccine discussions	Use of specific language when a caregiver declines COVID-19 vaccination that emphasizes their autonomy and maintains rapport to discuss vaccination at a later visit. (e.g., “Thank you for discussing the COVID-19 vaccine with me. It is certainly your choice, and I am happy to talk more about it in the future.”)
Motivational interviewing (MI) Skills	
Using **open-ended questions** to explore sources of hesitancy	Open-ended questions allow for a wide range of possible answers to allow for targeting counseling (e.g., “Tell me more about what makes you want to pass on getting your child the COVID-19 vaccine today.”)
**Reflecting** on patient statement	Conveying understanding of something that a caregiver or patient has said (e.g., “You are worried that the COVID-19 vaccine may have bad side effects.”)
**Asking permission**	Asking permission to provide information (e.g., “Is it okay if I tell you more about the side effects that can occur with the COVID-19 vaccine?”)
Use **affirmative statements**	Emphasize a family’s strengths and skills (e.g., “I appreciate how dedicated you are to keeping your child healthy.”)

VICTORI, Virtual Immersive Communication Training on Recommending Immunization.

### Intervention mapping step 4

**FIG. 2.**
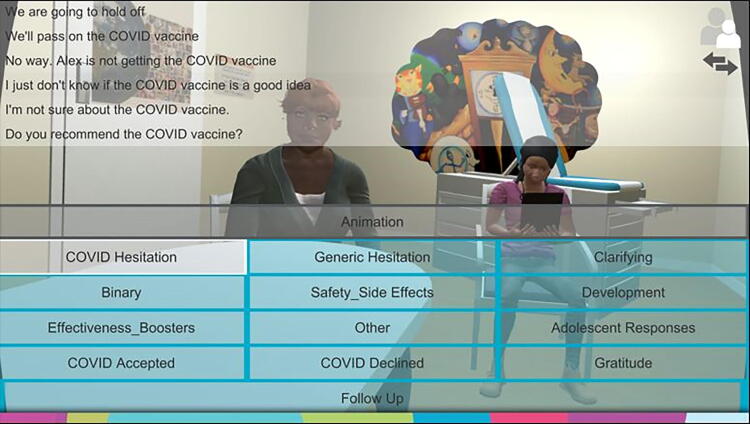
The human facilitator drives the actions of the graphical characters in real-time, including their verbal and non-verbal language in response to the user’s counseling. The facilitator dashboard provides access to prerecorded verbal language and body animations.

Outputs from this work included the final development of VICTORI-COVID-19 through IM step 4 (program production). It was produced using the Unity gaming platform with C# scripting.^40^ The speech for the graphical characters was created using Revoicer.^53^ VICTORI-COVID-19 includes four unique VR simulations to allow deliberate practice addressing the community-identified salient COVID-19 vaccine concerns and practicing the preferred MI communication skills. All scenarios took place during routine well-child care for an adolescent patient. Scenario 1 focuses on introducing the vaccine with no specific sources of hesitancy reported. Scenario 2 addresses safety and short-term side effect concerns. Scenario 3 addresses concerns related to rapid development of the vaccine and concerns for long-term side effects (i.e., fertility concerns). Scenario 4 focuses on effectiveness concerns and the need for boosters. The flowsheets for each scenario are structured in such a way to positively and negatively reinforce behaviors based on the user input through verbal and nonverbal language by the graphical character. (Supplementary Materials 1) For example, if a user presumes that a family is agreeable to the COVID-19 vaccine without first assessing their interest in vaccination (as recommended by the planning board members), the graphical character will look away from the user and say, “No way. Alex is not getting the COVID-19 vaccine.” The intervention is designed for a clinician to complete independently and is human-facilitated (Fig. 2) so that a clinician can receive coaching following each scenario. This also allows for corrective feedback on less preferred behaviors, such as overconfidence during counseling. Brief didactic information is included between scenarios to highlight planning board communication preferences. The program objectives (IM step 2) were scaffolded to increase in complexity over the four scenarios and introduced by the facilitator, who provides the exemplar language developed and reviewed by the planning board. The intervention takes approximately 40 min to complete. It can be delivered via a 3D-mounted headset (compatible with all oculus and meta headsets supported by the Meta Quest application^54^) or via a screen using a teleconferencing platform (Supplementary video). The program can be run on any VR-capable computer with preferred specifications of a 2070 or better GPU and 16 Mb of RAM.

### Intervention mapping steps 5–6

Next, the team utilized a mixed-methods approach to complete IM steps 5 and 6^31^ to evaluate the effectiveness and implementation of the intervention via a pilot study at the medical center’s adolescent medicine primary care clinic. Analysis and dissemination of this pilot study are ongoing. Study progress and results have since been reported back in-person to the community planning board through two follow-up presentations.

## Discussion

Through IM, we were able to engage and codesign a VR intervention focusing on COVID-19 vaccine hesitancy with Black pediatric families and clinicians. We used surveys, focus groups, videos, and storyboarding to establish consensus among planning board members on a complex topic that had been understudied at the time. Our approach aligned with the Vancouver Statement 2015 and the Accreditation Council for Continuing Medical Education recommendation that training incorporate patients’ autonomous and authentic voices to ensure that care delivery is patient-centered.^55,56^ This approach serves as a model for others seeking to develop customized, culturally-relevant extended reality interventions.

Developing providers’ communication skills in MI to address sources of vaccine hesitancy while also respecting and maintaining the patients and families’ autonomy requires deliberate practice of skills in a safe environment. VR provides a method to allow for such deliberate practice and accelerate competencies related to MI, which has proven challenging to train providers in through traditional educational strategies such as lectures and workshops.^29,30,39^ Indeed, such workshops often demonstrate immediate attitude change, though do not typically produce sustained behavior change related to MI.^57,58^ Though screen-based VR simulation may support decreased spatial presence and immersion compared to use of a 3D-mounted headset, it still allows providers to suspend their disbelief easily in a familiar clinical environment to support the practice of specific communication competencies.^59^ The IM process allowed stakeholders to provide input on VR storyboarding that included providing feedback on the scenario flowsheets, the verbal and nonverbal language of the graphical characters, and the presenting physical characteristics of the graphical environment and characters. As such, beyond allowing for deliberate practice of skills, the use of VR allows investigators to obtain input on nuanced details of the training experience that will be consistently applied across providers. Thus, VR is a particularly advantageous educational strategy as it enables repeatable practice and standardization across learners allowing for assessment of objective performance metrics.

Barriers to scalability of the VR intervention across centers may include hardware costs, internet bandwidth, and the need for technical support. As such, screen-based delivery of the intervention to remote locations via teleconferencing software may support equitable access to such high-quality education, aiming to enhance COVID-19 vaccine rates among patients. Train the trainer models, in which initial adopters undergo facilitator training to educate local staff, are a potential approach to support widespread dissemination. Identification of how to best incorporate VR training into clinical workflows or protected educational time will be necessary to support its effective implementation and sustainability over time. Artificial intelligence (AI) applications may support intervention dissemination by removing the need for human facilitation. Thus, recording and transcribing user interactions with graphical characters during scenarios may support the development of such AI models. Such data should be stored on a secure database with transcription occurring in a timely manner to allow deletion of recordings that contain identifying information of the user (e.g., face or voice). However, future work is needed to understand how automating such VR training may impact the intervention’s acceptability among learners as well as its effectiveness.

This work had several limitations. It was a single-site design taking place in an urban setting that limits the generalizability of the findings. It is also important to note that community planning board members were mostly females. Next steps may include recruiting male caregivers and adolescents to further explore their insights and testing the intervention in various regions to capture more diverse perspectives. To establish the effectiveness of the intervention, a future multisite trial should be conducted in diverse geographical regions and obtain feedback from local caregivers and clinicians to ensure aligned perspectives with the scenario content or identify areas for adaptations. Outcomes of interest may include assessing vaccination rates as well as clinicians’ fidelity to the behavioral skills included in the VR intervention through simulation, direct observation, or recorded patient encounters that are coded using instruments such as the Motivational Interviewing Treatment Integrity tool with established validity evidence.^60,61^ It will also be important to obtain survey information from parents regarding their perspectives on clinicians’ COVID-19 counseling.

## Conclusions

Using IM resulted in an intervention centered on the needs and preferences of Black families. This work continues to highlight the importance of establishing and maintaining community partnerships to meet the needs and preferences of community members served in clinical practices. Though time and resource-intensive, such approaches are critical when cocreating interventions with communities to ensure patients and families’ autonomous and authentic voices are centered in care delivery.

## Abbreviations Used

AI: Artificial intelligence
COVID-19: Coronavirus disease 2019
HPV: Human papillomavirus
IM: Intervention Mapping
MI: Motivational Interviewing
U.S.: United States
VICTORI: Virtual Immersive Communication Training on Recommending Immunizations
VR: Virtual Reality
WE-CRAB: West End Community Research Advisory Board
