# Supplementary material for: Using Intervention Mapping to Develop a Virtual Reality Intervention to Support COVID-19 Vaccination Among Black Families
Source: J Med Ext Real. 2025 Sep 12;2(1):230–43. doi: 10.1177/29941520251377072 (PMC13387259; doi:10.1177/29941520251377072)
Supplement: Supplementary file 1 [file 29941520251377072_supplementary_data.pdf]

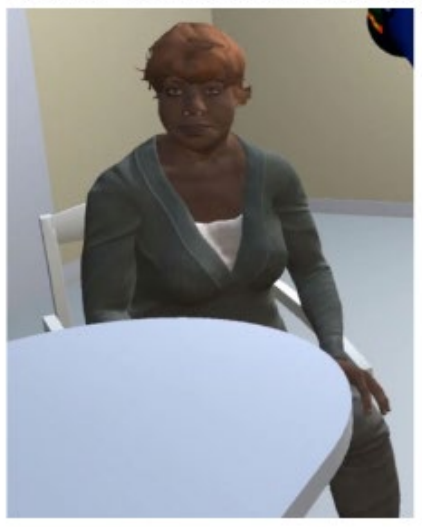

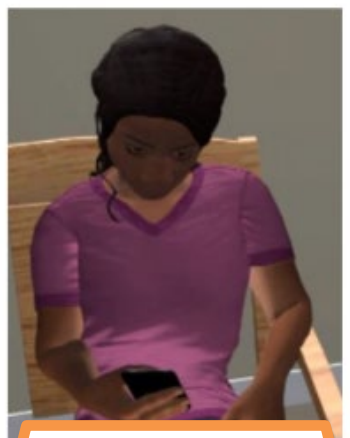

Hold IPad

SCENARIO 1: You are seeing a previously healthy 13-year-old female patient in clinic named Alex for her annual visit. Everything went well with her visit, and next you are planning to talk with Alex's Mom (Phyllis) about the COVID-19 vaccine. You have already met Phyillis so can go ahead and talk about the shot when you are ready.

- [ ] Participatory approach
- [ ] Presumptive announcement

↓

Legs Crossed

"We'll pass on the COVID Vaccine."

- [ ] Open-ended question
- [ ] Closed-ended question

↓

"I just don't know if the COVID-19 vaccine is a good idea"

- [ ] Recommendation
- [ ] No recommendation

↓

"I'm just not ready yet."
"Can I think about it more?"

- [ ] Respectfully accepts decision & leaves door open
- [ ] Affirmative statements

↓

"We'll talk about it at home and schedule a visit if we decide to get it."

SUCCESS

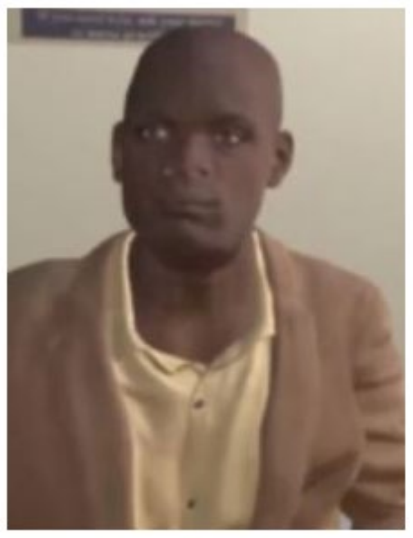

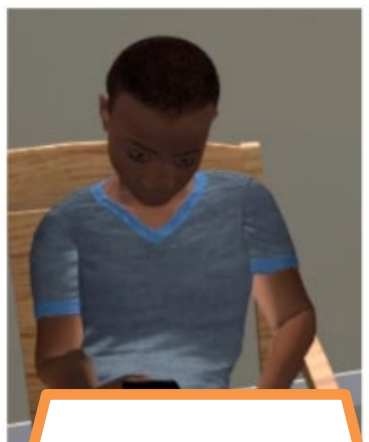

Hold IPad

SCENARIO 2: You are seeing a previously healthy 13-year-old male patient in clinic named Alex for his annual visit. Everything went well with his visit, and next you are planning to talk with Alex's Dad (Phil) about the COVID-19 vaccine. You have already met Phil so can go ahead and talk about the shot when you are ready.

☐ Participatory approach → *"No way. Alex is not getting the COVID vaccine."*

"We'll pass on the COVID Vaccine."

☐ Open-ended question → *"No way. Alex is not getting the COVID vaccine."*

Looks Away

Legs Crossed

"I heard the vaccine can get you sick"
"I don't want Alex to miss school"

☐ Recommendation → *"Do you recommend the COVID vaccine?"*

☐ Asking Permission

"Sure"

☐ Provides Information

"I don't know still"

☐ Open-ended question

"We're not interested in discussing it."
"Could we get our discharge paperwork?"

VISIT ENDS

"Can't the vaccine potentially cause problems with your heart?"

☐ Provides Information

☐ Respectfully accepts decision & leaves door open

SUCCESS

"Thank for the information…"
"I need to do more research on it."

→ *"That sounds good."*

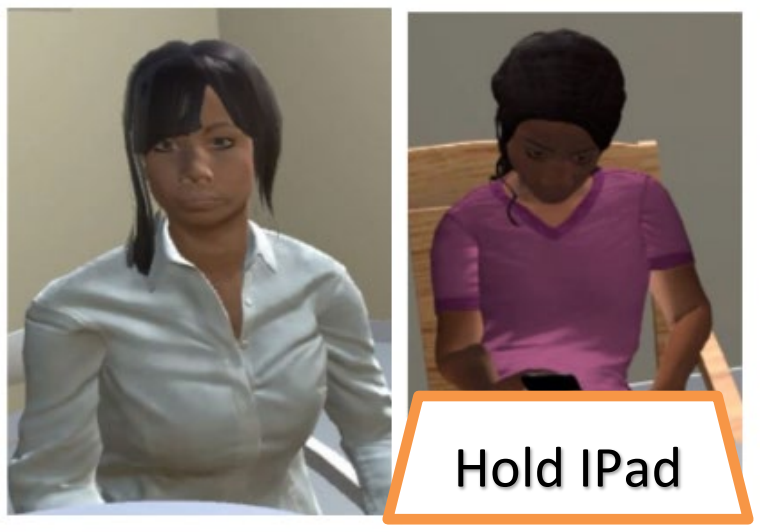

SCENARIO 3: You are seeing a previously healthy 13-year-old female patient in clinic named Alex for her annual visit. Everything went well with her visit, and next you are planning to talk with Alex's Mom (Phyllis) about the COVID-19 vaccine. **She is also due for 11-year old vaccines including: Tdap, HPV, and Menactra.** You have already met Phyllis so can go ahead and talk about the shots when you are ready.

☐ Presumptive → *"No way. Alex is not getting the COVID vaccine."* → VISIT ENDS

☐ Participatory approach

"We'll pass on the COVID Vaccine."

☐ Open-ended question → *"No way. Alex is not getting the COVID vaccine."* → VISIT ENDS

Looks Away

Legs Crossed

"The vaccine just came out of nowhere. It was developed too fast…LONG statement"

☐ Avoids interrupting

☐ Reflection

☐ Recommendation → *"Do you recommend the COVID vaccine?"* → VISIT ENDS

☐ Asking Permission

"Sure"

☐ Provides Information

"I still really don't know"

☐ Open-ended question

☐ Low Performance → *"I'm not ready yet." "We'll keep thinking about it"*

VISIT ENDS

"We're not interested in discussing it." "Could we get our discharge paperwork?"

"Who knows what could happen in 10-years from this vaccine" "I'm worried this might impact her ability to have children in the future."

☐ High Performance re: MI behaviors

Cues

Advises on timing of second dose ☐

SUCCESS

"You know what. I do want to keep Alex as healthy as possible." "The COVID vaccine does seem like a good idea."

"Thank you for the info." "This was helpful."

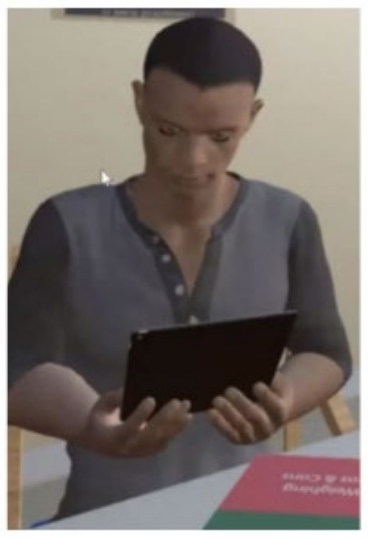

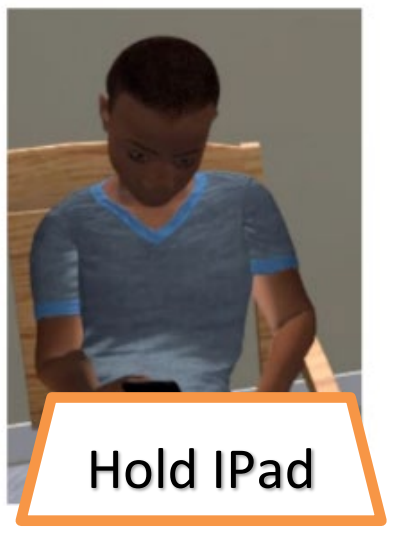

Hold IPad

SCENARIO 4: You are seeing a previously healthy 13-year-old male patient in clinic named Alex for his annual visit. Everything went well with his visit, and next you are planning to talk with Alex's Dad (Phil) about the COVID-19 vaccine. **He is also due for 11-year old vaccines including: Tdap, HPV, and Menactra.** You can go ahead and talk with Phil about the shots when you are ready.

- Presumptive → *"No way. Alex is not getting the COVID vaccine."*
- Participatory approach

"We'll pass on the COVID Vaccine."

- Open-ended question

"It seems like even people that go the vaccine are getting infected." "What's the point?"

Legs Crossed

- Reflection
- Recommendation → *"Do you recommend the COVID vaccine?"*
- Asking Permission

"Sure"

Legs Crossed

- Provides Information

"I still really don't know"

- Open-ended question

"I don't get why we need to get boosters?"

- Provides Information
- Shares Experiences

"Did you get a booster?" "Did you have side effects?" → *Acceptance statement* → Advises on timing of second dose (*Cues*) → **SUCCESS**

"We're not interested in discussing it." "Could we get our discharge paperwork?"

VISIT ENDS
